# *Lactobacillus plantarum* And Inulin: Therapeutic Agents to Enhance Cardiac Ob Receptor Expression and Suppress Cardiac Apoptosis in Type 2 Diabetic Rats

**DOI:** 10.1155/2020/4745389

**Published:** 2020-05-15

**Authors:** Safa Sefidgari-Abrasi, Pouran Karimi, Leila Roshangar, Mohammad Morshedi, Khadijeh Bavafa-Valenlia, Maryam Saghafi-Asl, Sara Mohiti, Marziyeh Rahimiyan-Heravan

**Affiliations:** ^1^Student Research Committee, Tabriz University of Medical Sciences, Tabriz, Iran; ^2^Drug Applied Research Center, Tabriz University of Medical Sciences, Tabriz, Iran; ^3^Nutrition Research Center, School of Nutrition and Food Sciences, Tabriz University of Medical Sciences, Tabriz, Iran; ^4^Neurosciences Research Center, Tabriz University of Medical Sciences, Tabriz, Iran; ^5^Stem Cell Research Center, Tabriz University of Medical Sciences, Tabriz, Iran; ^6^Department of Clinical Nutrition, School of Nutrition and Food Science, Tabriz University of Medical Sciences, Tabriz, Iran

## Abstract

**Background:**

T2DM may cause increased levels of oxidative stress and cardiac apoptosis through elevated blood glucose. The present study investigated the effects of *Lactobacillus plantarum* (*L*. *plantarum*) as a probiotic strain and inulin as a prebiotic supplement on cardiac oxidative stress and apoptotic markers in type 2 diabetes mellitus (T2DM) rats.

**Methods:**

A high-fat diet and a low dose of streptozotocin were used to induce type 2 diabetes. The rats were divided into six groups which were supplemented with *L*. *plantarum*, inulin, or their combination for 8 weeks.

**Results:**

The results showed improved activity of cardiac antioxidant parameters including total antioxidant capacity (TAC), superoxide dismutase (SOD), and glutathione peroxidase (GPx) (*P* < 0.001, *P* < 0.01, and *P* < 0.01, respectively) and decreased level of cardiac malondialdehyde (MDA) concentration (*P* < 0.05). These changes were accompanied with increased protein expression of cardiac obesity receptor (Ob-R) (*P* = 0.05) and reduced apoptotic markers such as tumor necrosis factor-alpha (TNF-*α*), Fas ligand (FasL), and caspase proteins (*P* < 0.001, *P* = 0.003, and *P* < 0.01, respectively) in T2DM rats after concurrent *L*. *plantarum* and inulin supplementation. Moreover, a remarkable correlation of cardiac Ob-R and oxidative stress parameters with cardiac apoptotic markers was observed (*P* < 0.01).

**Conclusion:**

The concurrent use of *L*. *plantarum* and inulin seems to be beneficial, as they can lead to decreased heart complications of T2DM via reducing cardiac apoptotic markers.

## 1. Introduction

Diabetes is a prevalent and progressive metabolic disorder. Approximately, 1 in 11 adults suffers from this disease worldwide and over 90% of diabetic patients are type 2 diabetes mellitus (T2DM) [1]. The risk of death and cardiovascular complications among diabetic patients is 2 to 4 times higher than healthy individuals [2]. Diabetic cardiomyopathy (DCM) is one of the serious complications of diabetes identified by structural and functional deficits in the heart such as cardiac tissue fibrosis and cardiomyocyte apoptosis [3, 4]. Apoptosis has been considered as a result of diabetic hyperglycemia, hyperlipidemia, inflammation, and endoplasmic reticulum (ER) stress in the cardiomyocytes of the diabetic heart [5]. In comparison to the nondiabetic individuals, diabetic individuals show an 85-fold increase in cardiomyocyte apoptosis [6].

Oxidative stress is a key factor for apoptosis and diabetic cardiomyopathy. The balance between the production of reactive oxygen species (ROS) and their elimination through antioxidants production is essential for the maintenance of cardiovascular health [7]. According to Tangvarasittichai [8], hyperglycemia leads to increased ROS generation of which ROS-induced oxidative stress may cause abnormal gene expression, defective signal transduction, and apoptosis of the cardiomyocytes [9]. Diabetes reduces the cardiac activity of superoxide dismutase (SOD) and glutathione peroxidase (GPx) as well as impairs SOD expression [10, 11]. Guo et al. [12] revealed the increased formation of malondialdehyde (MDA) and reduced activity of SOD in the cardiomyocytes exposed to a high-glucose concentration. The cardiac protein expression of caspase-9, caspase-8, and caspase-3 can increase in diabetic rats. This would lead to hyperglycemia-induced apoptosis initiated by ROS derived from high glucose levels [13]. ROS leads to the initiation of tumor necrosis factor-alpha (TNF-*α*) gene expression [14]. On the other hand, TNF-*α* causes ROS production and contributes to tissue fibrosis in the diabetic state [13].

ROS-induced TNF-*α* expression may cause cardiomyocyte apoptosis through the upregulation of the Fas expression in high-glucose situations [15, 16]. The role of the Fas/Fas ligand (FasL) apoptotic pathway that leads to the activation of caspase cascade has been revealed in streptozotocin- (STZ-) induced apoptosis [17]. It was demonstrated that the production of the proinflammatory cytokines can be reduced by probiotic supplementation [18]. Sadeghzadeh et al. [19] also reported that TNF-*α* and oxidative stress damage can be suppressed through probiotic supplementation in a rat myocardial infarction model.

Probiotics are known as live microbial food supplements or bacteria components that modify the composition of the oral and enteric microflora, reduce adipose cell size, and promote leptin/adiponectin ratio [20]. According to Castex et al. [21], a probiotic diet might lead to increased activities of some antioxidant enzymes and reduced oxidative stress. Prebiotics are defined as nondigestible food ingredients including dietary fibers that promote the growth of beneficial bacteria in the gut [22]. Inulin is a prebiotic which can mitigate oxidative status by reducing lipid peroxidation and upregulating gene expressions of various antioxidant enzymes in different tissues [23]. Moreover, inulin supplementation could significantly improve leptin sensitivity [24]. Synbiotic is a combination of probiotic and prebiotic that may be beneficial in the treatment of diabetes through modulating serum insulin levels [25]. A significant increase of SOD, GPx, and catalase (CAT) was observed after concurrent administration of *Lactobacillus casei* and inulin among healthy volunteers [26]. Bejar et al. [27] also reported anti-diabetic effects of *L*. *plantarum* through the inhibition of *α*-glucosidase activity which can decrease fasting and postprandial blood glucose, in addition to insulin resistance and oxidative stress [28]. Furthermore, *L*. *plantarum* could increase serum levels of leptin [29] which may suppress heart apoptosis [30].

Diabetes leads to weight loss and increased food intake due to reduced concentration of serum leptin [31]. Leptin promotes heart function by improving glucose and fatty acid metabolism and reducing heart apoptosis [32]. It may also be associated with decreased apoptosis and fibrosis via activating SOD [33]. In addition, leptin reduces apoptosis in beta cells at physiological concentrations, by regulating BCL-2 and Bax expression [34].

Due to the important effects of the probiotic and prebiotic supplementation on hyperglycemia-induced oxidative stress and its protective impact on the production of proinflammatory cytokines, this paper investigated the effects of separate and concurrent supplementation of *Lactobacillus plantarum* 1085 (ATCC 8014) and inulin on T2DM-induced cardiac apoptosis by evaluating the cardiac antioxidant parameters including MDA, total antioxidant capacity (TAC), GPx, and SOD activity and the expression of the cardiac obesity receptor (Ob-R) as well as cardiac apoptotic markers. In addition, the correlations of these antioxidant enzymes and cardiac Ob-R with protein expression of cardiac TNF-*α*, FasL, and caspase were examined in T2DM male rats.

## 2. Materials and Methods

### 2.1. Animal Preparation and Experimental Protocol

The study was carried out on 35 male Wistar rats (200 ± 20 gr and 6 (±1) week old) purchased from the Laboratory Animal Center of Tabriz University of Medical Sciences, Tabriz, Iran. All rats were housed in a temperature- and humidity-controlled room at 22-25°C on 40-60% (four rats per cage). The rats were maintained at 12 : 12 h light/dark cycle and allowed *ad libitum* access to food and water.

### 2.2. Diet and Induction of T2DM

After the adaptation of the animals to the new environment through maintenance under a normal diet for 7 days, the rats were randomly assigned into six groups, as follows: HC: healthy control (*n* = 6); DC: diabetic control (*n* = 6); DSh: diabetic sham (*n* = 6); DL: diabetics treated by *L*. *plantarum* (*n* = 6); DI: diabetics treated by inulin (*n* = 5); DLI: diabetics treated by *L*. *plantarum* and inulin (*n* = 6). Normal diet contained 12% fat, 22% protein, and 66% carbohydrate. A high-fat diet (HFD) consisted of 58% fat, 17% protein, and 25% carbohydrate of which the composition is described in Table 1. The healthy control group continued to receive a normal diet until the end of the study (Table 2). To induce T2DM, the other groups were fed HFD for 4 weeks (from week 2 to week 5). Then, the HFD-fed rats received a single low dose of STZ (35 mg/kg intraperitoneally) in 50 *μ*L citrate buffer (0.1 M, pH: 4.5) [35, 36]. Three days after injection, they were grouped to begin the intervention. After 12 h fasting, the rats with fasting blood sugar (FBS) of 250 mg/dL and higher were supposed as diabetic rats. After STZ induction, all of the control and diabetic rats were fed with the normal diet until the end of the study (week 6 to 13). In addition, all intervention groups received either probiotic or prebiotic for 8 weeks (week 6 to 13).

### 2.3. Preparation of the Supplementation

*L*. *plantarum* ATCC 8014 was acquired from the Biotechnology Research Center, TBZMED, Iran. *L*. *plantarum* was inoculated in Man-Rogosa-Sharpe (MRS) broth and cultured in aerobic conditions. The suspension was freshly prepared at a concentration of 10^7^ colony-forming units per milliliter (CFU/mL) within 8 weeks. All rats received the suspension, using a gastric gavage once a day for 8 weeks [37]. The inulin content of the rat diet was assigned based on 5% of the daily food weight and was dissolved in drinking water.

### 2.4. Anesthesia and Tissue Extraction

At the end of the administration, after 12 h fasting, the animals were anesthetized with sodium pentobarbital (65 mg/kg BW IP, Sigma). Next, the left ventricle samples were hemogenized with 1 mL PBS and centrifuged for 40 min at 12000 rpm at 4°C. Then, the supernatant was separated and transferred into the microtubule and stored at −80°C for more experimental analysis.

### 2.5. Biochemical Assays

Cardiac levels of oxidative stress indices including SOD, GPx, and TAC were measured by biochemical kits, following the manufacturer's instruction (Randox Company, England). TAC was determined by Randox's total antioxidant status kit, as described by Miller et al. [38]. Cardiac SOD was measured, according to Breinholt et al., using a RANSOD kit (Randox Labs Crumlin, UK) [39]. Also, cardiac GPx was assayed by a RANSEL Kit (Randox Labs Crumlin, UK), as described by Paglia and Valentine [40]. Thiobarbituric acid was used to measure the MDA level; TBARS were determined by Esterbauer and Cheeseman [41]. In addition, serum glucose was evaluated spectrophotometrically using a diagnostic reagent kit (Pars Azmoon Company, Tehran, Iran). Furthermore, serum concentration of insulin was determined by the ELISA method (Shanghai Crystal Day Biotech Co., Ltd., China).

### 2.6. Western Blot Analysis

SDS-PAGE was performed by 10% polyacrylamide gels. 10 *μ*L of each sample was electrophoresed at 100v and proteins were transferred to polyvinylidene difluoride (PVDF) membranes, using a transfer buffer and a Bio-Rad Scientific Instruments Transfer Unit (Sigma-Aldrich). PVDF membranes were incubated overnight at 4°C with primary antibodies against both the full-length and the cleaved fragments of caspase-9, caspase-8, and caspase-3 (Cell Signaling Technology) as well as against Ob-R, TNF-*α*, and FasL (Santa Crus Biotechnology) at a ratio of 1 : 200 for all of the first antibodies. This process was followed by incubation with anti-rabbit or anti-mouse secondary antibodies (Santa Crus Biotechnology) at the 1 : 5000 ratio. Blots were washed and inserted in the ECL substrate solution for 30 seconds, according to the manufacturer's instruction. The immunoblots were probed with an antibody recognizing *β*-actin as an internal control.

### 2.7. Statistical Analysis

All data were presented as the means ± SD for rats in each group. Statistical analyses were carried out by SPSS statistics software (version 25). Drawing of graphs and charts were performed, using the STATA (version 14) and GraphPad Prism (Version 8), respectively. One-way analysis of variance (ANOVA), followed by Tukey's test as a *post hoc* analysis, was utilized to examine the level of significance between groups. Correlations between two variables were carried out, using the Pearson correlation coefficient test. *P* < 0.05 was regarded as statistically significant.

## 3. Results

### 3.1. Effects on Food Intake, FBS, and Serum Insulin

**Figure 1:**
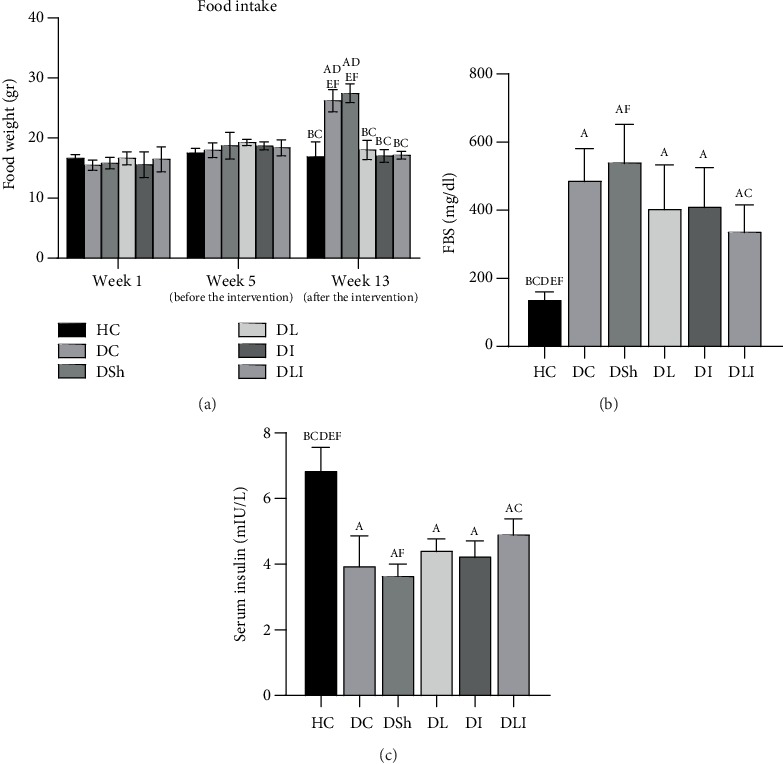
Effects of *L*. *plantarum* and inulin supplementation on (a) food intake, (b) FBS, and (c) serum insulin of the control and diabetic rats (*n* = 35). HC: healthy control; DC: diabetic control; DSh: diabetic sham; DL: diabetics treated by *L*. *plantarum*; DI: diabetics treated by inulin; DLI: diabetics treated by *L*. *plantarum* and inulin. Data were expressed as mean ± SD and regarded significantly different at *P* < 0.05 with a *post hoc* Tukey test.

As presented in Figure 1, food intake was significantly increased in the DC rats, in comparison to the HC group (*P* < 0.001). Also, there was a significant reduction of food intake in the treatment groups, compared with the DSh rats (*P* < 0.001). As compared to the HC group, a significant increase of FBS was observed in the DC rats as well as a remarkable decrease in serum insulin (*P* < 0.001). In addition, in comparison to the DSh rats, there was a significant reduction of FBS in the DLI group as well as a great increase in serum insulin (*P* = 0.01 and *P* = 0.005, respectively). Moreover, there was a strong correlation of food intake with cardiac apoptotic markers in the last week of the study as well as a great correlation of FBS and serum insulin with cardiac apoptotic proteins (Table 3).

### 3.2. Effects on the Cardiac TAC, SOD, and GPx

**Figure 2:**
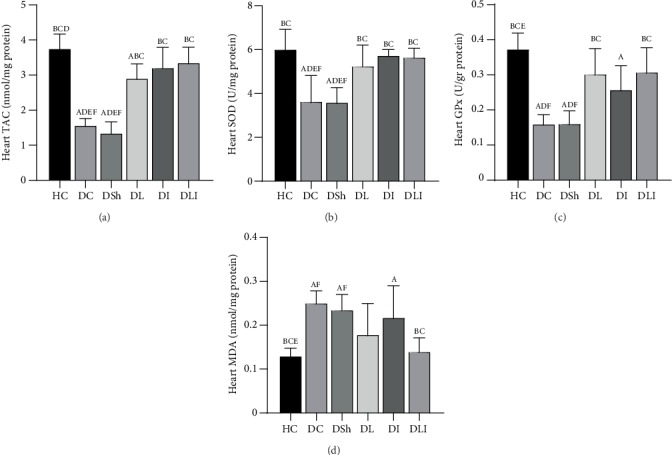
Effects of *L*. *plantarum* and inulin supplementation on oxidative stress markers in the left ventricles of excided hearts from the control and diabetic rats (*n* = 35). (a–d) The level of TAC, SOD activity, GPx activity, and MDA concentration in the left ventricles of the rats, respectively. HC: healthy control; DC: diabetic control; DSh: diabetic sham; DL: diabetics treated by *L*. *plantarum*; DI: diabetics treated by inulin; DLI: diabetics treated by *L*. *plantarum* and inulin. Data were expressed as mean ± SD and regarded significantly different at *P* < 0.05 with a *post hoc* Tukey test.

**Figure 3:**
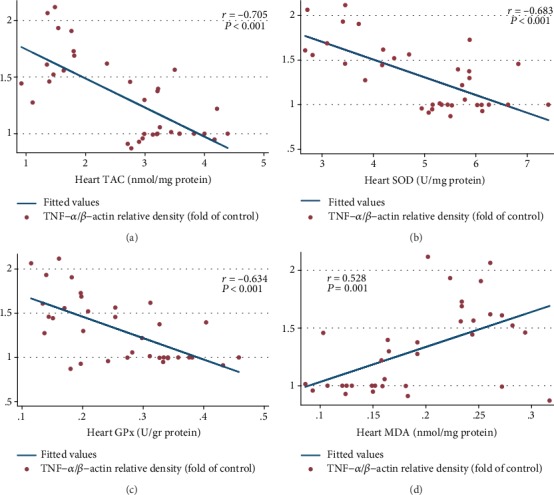
Inhibiting impacts of *L*. *plantarum* and inulin administration on the TNF-*α* expression in the left ventricles of the rats through the promotion of the cardiac oxidative status. (a–d) Correlation of the cardiac TAC, SOD activity, GPx activity, and MDA concentration with protein expression of TNF-*α*, respectively (*n* = 35). Correlation between two variables was determined, using the Pearson correlation coefficient; *P* < 0.05 was considered statistically significant.

As compared to the HC group, there was a significant decrease in the cardiac TAC, SOD, and GPx in the DC rats (*P* < 0.001). In comparison to the DSh group, there was a significant increase of the cardiac TAC, SOD, and GPx in the DL (*P* < 0.001, *P* = 0.013, and *P* = 0.001, respectively) as well as the DLI (*P* < 0.001, *P* = 0.001, and *P* = 0.001, respectively) group. In addition, the cardiac TAC and SOD increased significantly in the DI rats, compared to the DSh group (*P* < 0.001 and *P* = 0.001, respectively). In contrast, insignificant differences were observed in the cardiac GPx of the DI rats (Table 4, Figure 2). Also, cardiac TAC, SOD, and GPx were significantly correlated with cardiac apoptotic markers (Table 5). Furthermore, there was a strong correlation of the cardiac TAC, SOD, and GPx with cardiac TNF-*α* expression (*P* < 0.001, *P* < 0.001, and *P* < 0.01, respectively) (Figure 3).

### 3.3. Effects on the Cardiac MDA Concentration

Cardiac MDA concentration significantly increased in the DC group, compared with the HC rats (*P* = 0.001). There was no significant decrease in the cardiac MDA concentration of the DL and DI groups, compared with the DSh rats after supplementation. In contrast, *post hoc* analyses revealed a significant decrease of the cardiac MDA concentration in the DLI group, in comparison to the DSh rats (*P* = 0.013). However, the test showed insignificant differences in the cardiac MDA concentration among the treatment groups (Table 4, Figure 2). The Pearson correlation test showed a strong correlation between the cardiac MDA concentration and apoptotic markers (Table 5). In addition, a significant correlation was observed between the cardiac MDA concentration and TNF-*α* expression (*P* < 0.01) (Figure 3).

### 3.4. Effects on Cardiac Protein Expression of the Ob-R, FasL, and TNF-*α*

**Figure 4:**
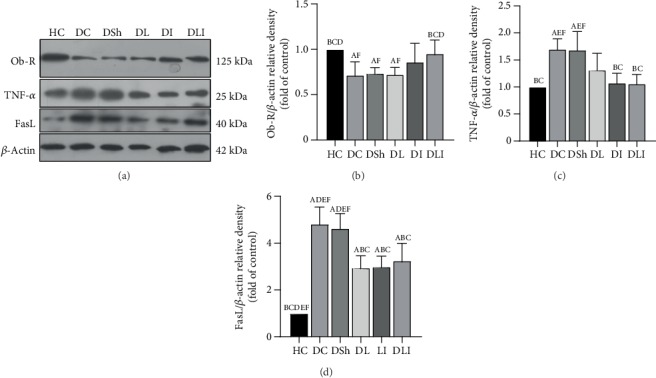
Effects of *L*. *plantarum* and inulin supplementation on the protein expression of Ob-R, TNF-*α*, and FasL in the left ventricles of excided hearts from the control and diabetic rats. (a) The protein levels of Ob-R, TNF-*α*, and FasL in the left ventricles of the rats, as determined by western blot analysis. (b–d) Relative protein quantification of Ob-R, TNF-*α*, and FasL on the basis of *β*-actin. HC: healthy control; DC: diabetic control; DSh: diabetic sham; DL: diabetics treated by *L*. *plantarum*; DI: diabetics treated by inulin; DLI: diabetics treated by *L*. *plantarum* and inulin. Values are mean ± SD and regarded significantly different at *P* < 0.05 with a *post hoc* Tukey test.

**Figure 5:**
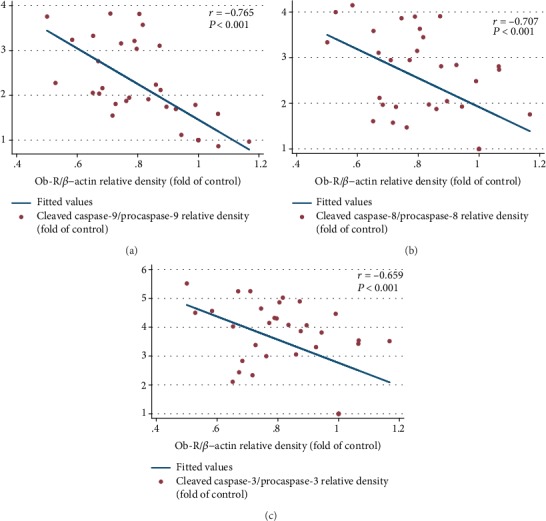
Inhibiting effects of *L*. *plantarum* and inulin supplementation on expression of the caspase proteins in the left ventricles of the rats through the increased expression of the Ob-R. (a–c) Correlation of the cardiac protein expression of the Ob-R with the cardiac expression of the activated caspase-9, caspase-8, and caspase-3, respectively (*n* = 35). Correlation between two variables was determined, using the Pearson correlation coefficient; *P* < 0.05 was considered statistically significant.

As compared with the HC group, there was a significant decrease of the Ob-R expression in DC rats (*P* = 0.005). Also, FasL and TNF-*α* protein expressions were significantly higher in the DC rats, in comparison with the HC group (*P* < 0.001). *L*. *plantarum* supplementation significantly decreased the cardiac protein expression of FasL, but not TNF-*α*, compared with the DSh group (*P* < 0.001). However, inulin supplementation decreased protein expression of FasL and TNF-*α*, compared with the DSh rats (*P* = 0.001 and *P* = 0.002, respectively). Such a decrease was also observed in the cardiac protein expression of FasL and TNF-*α* in the hearts of the DLI group, in comparison to the DSh rats (*P* = 0.003 and *P* < 0.001, respectively) (Figure 4). In addition, the increase of Ob-R expression in DLI rats was critically significant, compared to the DSh group (*P* = 0.05). In contrast, there was no significant increase in protein expression of the Ob-R in DL and DI rats (Figure 4). Moreover, a strong correlation was found between the cardiac TNF-*α* expression and apoptotic markers (Table 5) as well as a great correlation between the cardiac Ob-R expression and cardiac expression of the caspase proteins (Figure 5).

### 3.5. Effects on Protein Expression of Cleaved Caspase-9, Caspase-8, and Caspase-3 in the Hearts of the Control and Diabetic Rats

**Figure 6:**
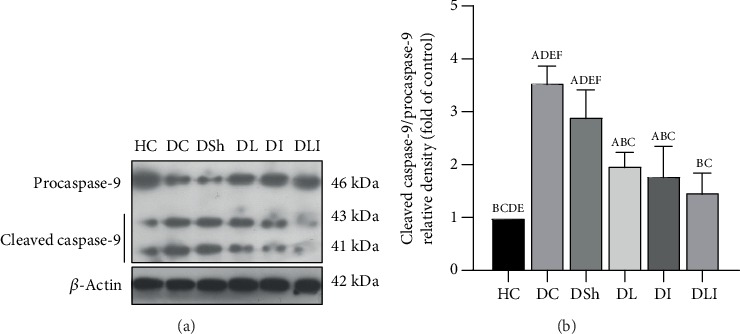
Effects of *L*. *plantarum* and inulin supplementation on protein expression of the cardiac caspase-9. (a) The protein level of caspase-9 in the left ventricles of excided hearts from the control and diabetic rats, as determined by western blot analysis. (b) Relative protein quantification of cleaved caspase-9, on the basis of procaspase-9. HC: healthy control; DC: diabetic control; DSh: diabetic sham; DL: diabetics treated by *L*. *plantarum*; DI: diabetics treated by inulin; DLI: diabetics treated by *L*. *plantarum* and inulin. Values are means ± SD and regarded significantly different at *P* < 0.05 with a *post hoc* Tukey test.

**Figure 7:**
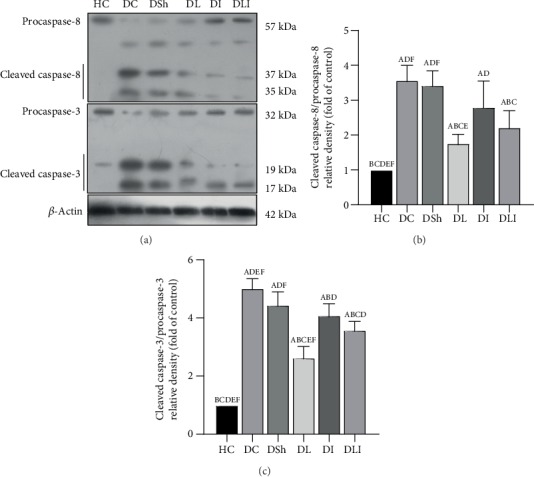
Effects of *L*. *plantarum* and inulin supplementation on protein expression of the cardiac caspase-8 and caspase-3. (a) The protein levels of caspase-8 and caspase-3 in the left ventricles of excided hearts from the control and diabetic rats, as determined by western blot analysis. (b) Relative protein quantification of cleaved caspase-8, on the basis of procaspase-8. (c) Relative protein quantification of cleaved caspase-3, on the basis of procaspase-3. HC: healthy control; DC: diabetic control; DSh: diabetic sham; DL: diabetics treated by *L*. *plantarum*; DI: diabetics treated by inulin; DLI: diabetics treated by *L*. *plantarum* and inulin. Values are means ± SD and regarded significantly different at *P* < 0.05 with a *post hoc* Tukey test.

Western blot analysis showed a significant increase in the protein expression of cleaved caspase-9, caspase-8, and caspase-3 in the DC rats, compared with the HC group (*P* < 0.001). In comparison to the DSh group, there was a significant reduction in the cardiac protein expression of cleaved caspase-9, caspase-8, and caspase-3 in the DL (*P* = 0.002, *P* < 0.001, and *P* < 0.001, respectively) as well as the DLI rats (*P* < 0.001, *P* = 0.001, and *P* = 0.002, respectively). Inulin supplementation caused a significant reduction in the protein expression of cleaved caspase-9, compared to the DSh rats (*P* < 0.001) (Figure 6). However, there were no significant differences in the cardiac protein expression of cleaved caspase-8 and caspase-3 in the DI rats, in comparison with the DSh group (Figure 7). In addition, the protein expression of cleaved caspase-3 was significantly lower in the DL rats, compared with the DI and DLI groups (*P* < 0.001). Also, there was a significant decrease in the protein expression of cleaved caspase-8 in the DL (but not DLI) rats, in comparison to the DI group (*P* = 0.006) (Figure 7).

## 4. Discussion

To our knowledge, the results of the present study demonstrated inhibiting effects of *L*. *plantarum* and inulin supplementation on the cardiac apoptotic markers by increasing the expression of Ob-R and antioxidant parameters in the diabetic rat hearts for the first time. DCM is one of the main heart complications in T2DM which increases the risk of heart failure and mortality in diabetic patients [42]. Myocardial cell apoptosis occurring in the DCM may play an important role in the development of the disease [43].

In the present study, the levels of antioxidant enzymes were evaluated in the cardiac tissue. Similar to previous researches [44, 45], we observed a significant reduction of cardiac TAC, GPx, and SOD activities and a remarkable increase of the cardiac MDA in the T2DM rats which were reversed after supplementation with *L*. *plantarum* and inulin.

Hyperglycemia condition in T2DM can lead to increased ROS production [8, 14] which upregulates TNF-*α* expression and induces cardiomyocyte apoptosis [46] via increasing the expression of the Fas [16]. Higher production of glucose-induced ROS plays an important role in the intrinsic apoptosis pathway by increasing cytochrome c release and activating caspase-9 and caspase-3 [47]. On the other hand, TNF-*α* provokes the activation of caspase-8 in the cardiomyocytes, leading to ROS generation [48] and subsequent apoptosis [49]. According to Wang et al. [50], antioxidant enzymes such as SOD are key factors in the reduction of the diabetes-induced cardiac apoptosis in mice. In a human research, Aouacheri et al. [44] illustrated decreased serum levels of GSH, GPx, and SOD activities as well as increased concentration of serum MDA in T2DM.

In the present research, increased activity of the cardiac SOD, GPx, and TAC was observed in the synbiotic group, accompanied by a reduced concentration of the cardiac MDA. Moreover, L. *plantarum* improved the cardiac activity of SOD, GPx, and TAC significantly. As described in our previous study by Morshedi et al. [51], there was also a considerable increase of serum TAC, SOD, and GPx activities and reduced concentration of serum MDA in the T2DM rats after a separate and concurrent supplementation of *L*. *plantarum* and inulin. However, in contrast with our results, Tunapong et al. [45] showed insignificant changes in the cardiac SOD by a separate and concurrent administration of the probiotics and prebiotics in the male obese insulin-resistant rats. But they revealed decreased cardiac MDA concentration in the rats treated by probiotics, prebiotics, and synbiotics. The reason for the observed difference seems to be due to the fashion by which diabetes was induced in the rats. The diabetic rats in our study were STZ induced, whereas those of the mentioned research were high-fat-diet-fed obese rats. Also, the composition of their supplement was different from the present study. We administered *L*. *plantarum* and inulin while they used *L*. *paracasei* and xylooligosaccharides in their study. Aluwong et al. [52] revealed the decreased concentration of serum MDA in *Saccharomyces cerevisiae*-supplemented diabetic rats with insignificant changes in the SOD activity. The observed controversy may be due to the type of diabetes or administered probiotic being studied. Unlike our research, Aluwong et al. studied on alloxan-induced T1DM rats supplemented with *Saccharomyces cerevisiae*. Moreover, their supplementation continued for four weeks which was shorter than ours. In a similar study by Zhang et al. [53], supplementation of *Lacobacillus casei* significantly reduced serum and hepatic levels of MDA and increased SOD and GSH-Px activities in hyperlipidemic rats. *In vivo* results of a research by Li et al. [54] indicated the ameliorative effects of *L*. *plantarum* C88 administration on the antioxidant status of the D-gal-induced oxidatively stressed mice including improved serum SOD activity and decreased concentration of hepatic MDA. In addition, Li et al. [28] revealed that *L*. *plantarum* X1 and L. plantarum CCFM30 can elevate SOD and GPx activities and ameliorate MDA level. Another similar study showed beneficial effects of *L*. *plantarum* BL0021 supplementation on hepatic and renal oxidative stress and apoptosis accompanied by reduced hepatic and renal MDA in pregnant rats [55].

In the present study, the western blot analysis revealed increased expression of TNF-*α* protein in the cardiomyocytes of the diabetic rats. As described above, TNF-*α* is an important mediator of the apoptotic pathway in the diabetic heart which stimulates the expression of the FasL protein. Mellado-Gil and Aguilar-Diosdado [17] demonstrated the role of the Fas/FasL system in the STZ-induced apoptosis in rats. Fas ligand provokes caspase-8 activation and causes cardiomyocyte apoptosis [56]. Similar to these studies, expression of the FasL was upregulated in the hearts of the T2DM rats in the present research. Also, there was a significant increase in the expression of caspase-9, caspase-8, and caspase-3 in the diabetic rats. In this regard, some studies demonstrated increased cardiac levels of the activated caspase-3 in the STZ-induced diabetic rats [57].

The results of the present research demonstrated reduced cardiac TNF-*α* and FasL expressions in the prebiotic and synbiotic groups. Additionally, it demonstrated a decreased expression of cleaved caspase-9, caspase-8, and caspase-3 in the hearts of the probiotic- and synbiotic-supplemented rats. Wang et al. [58] also reported anti-inflammatory and anti-apoptotic effects of *Lactobacillus paracasei* in the cardiomyocytes of the ovalbumin-induced allergic mice by decreasing the expression of cardiac TNF-*α*, caspase-3, and proapoptotic proteins such as Bax. In the present work, we also observed a strong correlation between the cardiac oxidative parameters and cardiac apoptotic markers which reveals the beneficial effects of the improved oxidative status on the prevention of cardiac apoptosis. Furthermore, there was a great correlation of the cardiac TNF-*α* expression with cardiac oxidative status and apoptotic proteins. A strong negative correlation of the cardiac TAC, SOD, and GPx with cardiac TNF-*α* expression was observed as well as a significant positive correlation between the cardiac MDA and TNF-*α* expression that indicates the inhibiting impacts of *L*. *plantarum* and inulin administration on TNF-*α* expression through the promotion of the cardiac oxidative status. However, the correlation of the cardiac apoptotic proteins with cardiac inflammatory and oxidative markers was not investigated in the mentioned previous studies.

In the present research, a high-fat diet and a single dose of STZ injection were used to develop hyperglycemia and insulin resistance [35, 36]. In fact, HFD was used to help induce T2DM before STZ injection [36]. Insulin resistance which occurs in type 2 diabetes mellitus is a situation that higher circulating insulin levels are necessary to achieve the integrated glucose-lowering response [59]. On the other hand, insulin secretion is severely impaired in poorly controlled type 2 diabetes which leads to the lower concentration of the serum insulin [60]. Impaired levels of insulin occur in diabetes which results in the disability of the cells to uptake glucose from the blood [29]. However, in the present study, the supplementation resulted in normalized levels of insulin and FBS in the intervention groups and prevented excessive food intake in the supplemented rats. In addition, as described in our previous study [29], serum leptin was significantly decreased in diabetic patients which was modulated after simultaneous administration of *L*. *plantarum* and inulin. In a similar study by Havel et al. [31], diabetes resulted in increased food intake by reducing circulating leptin levels. On the other hand, leptin inhibits the heart apoptosis by activating SOD or other possible manners [33]. Barouch et al. [30] demonstrated increased cardiac apoptosis in leptin-deficient ob/ob and leptin-resistant db/db mice. Moreover, McGaffin et al. [61] reported higher rates of caspase-3 activity and cardiac apoptosis as well as reduced expression of Bcl-2 in the infarcted tissue of lean and obese ob/ob mice, compared with the wild-type mice. Leptin is a survival cytokine for human neutrophils by inhibiting the activation of Bid, Bax, caspase-8, and caspase-3 and decreasing the mitochondrial release of cytochrome *c* [62]. Furthermore, Fruhbeck et al. [63] demonstrated that leptin reduces the expression of genes involved in inflammation and oxidative stress in the adipose tissue and skeletal muscle of ob/ob mice. Also, it has a protective effect against ethanol-induced oxidative stress [64].

**Figure 8:**
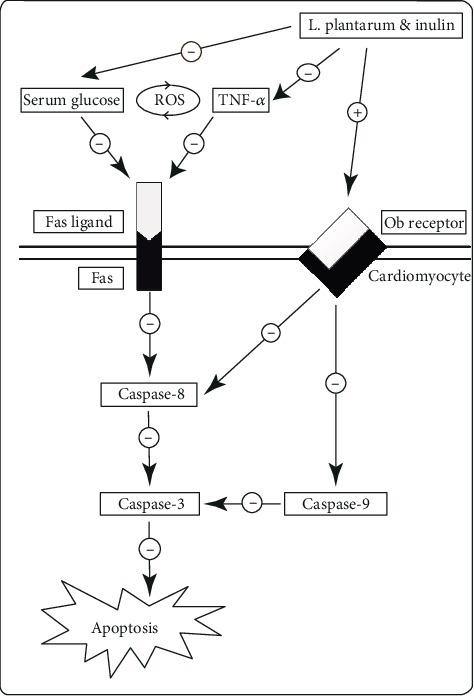
Suggested hypothesis of STZ-induced cardiac apoptosis, inhibited by *L*. *plantarum* and inulin supplementation in T2DM rats.

According to our results, a significant increase in cardiac Ob-R expression was observed by the simultaneous use of inulin and *L*. *plantarum*. Also, there was a strong positive correlation of food intake and serum glucose with cardiac apoptotic markers in the last week of the study along with a great negative correlation of serum insulin and cardiac Ob receptor expression with the activation of caspase proteins. These results indicate beneficial effects of inulin and *L*. *plantarum* supplementation on cardiac apoptosis through controlling food intake, FBS, serum insulin, and enhanced expression of cardiac Ob receptor in diabetes (Figure 8).

According to our previous work [29], improved lipid profile and metabolic status including modulated serum leptin can be achieved in T2DM rats by the administration of *L*. *plantarum* and inulin. This improved metabolic status and reduced blood glucose level can lead to decreased weight loss and food intake, followed by lower cardiac apoptotic markers observed in T2DM. *L*. *plantarum* and inulin supplementation may suppress cardiac apoptosis via direct and indirect mechanisms. *L*. *plantarum* and inulin supplementation could indirectly decrease apoptosis in diabetic hearts via improving the metabolic status and reducing serum glucose and food intake. In addition, it could inhibit cardiac apoptosis directly through different mechanisms such as the upregulated concentration of antioxidants and Ob-R as well as the downregulated expression of TNF-*α* in the cardiac tissue. Although no study has suggested the exact suitable dose of *L*. *plantarum* in order to reap benefits for cardiac disease, *L*. *plantarum* is safe at high doses. Fuentes et al. [65] illustrated that patients treated by daily consumption of *L*. *plantarum*-containing capsules (100 mg per capsule) for 12 weeks showed a significant reduction of LDL-C and improved other lipid parameters. Furthermore, *L*. *plantarum* administration leads to a reduction in cardiovascular disease risk factors such as systolic blood pressure, leptin, and fibrinogen in smokers consuming 400 mL/d of a rose-hip drink containing *L*. *plantarum* 299v (5 × 10^7^ CFU/mL) for 6 weeks [66].

In summary, *L*. *plantarum*, inulin, or their combination can modulate cardiac antioxidant enzymes which result in lower protein expression of the cardiac apoptotic markers. Moreover, stronger effects of the concurrent supplementation of *L*. *plantarum* and inulin than their separate administration were revealed. In addition to the useful effects of the supplements, the present study had some limitations. One of the major limitations of the present research was that we could not examine the microbial population directly due to financial and time constraints. Also, the duration of the supplementation was a bit shorter which was due to the consideration of the higher mortality rate of the animals, proposed to be tested over a longer period of time. In the present research, the effect of *L*. *plantarum* and inulin on other apoptotic pathways was not investigated which is suggested to be investigated in future studies. Furthermore, we did not study the possible effects of our research on female rats. More animal and human researches are recommended to evaluate the possible effects of our interventions on female rats, other species, and human subjects.

## 5. Conclusion

Our findings provided the first evidence that *Lactobacillus plantarum* 1085 (ATCC 8014) and inulin supplementation could lead to a significant decrease of TNF-*α*-induced cardiac apoptosis in T2DM. The results also revealed decreased expression of cardiac apoptotic proteins including TNF-*α*, FasL, and caspase proteins after supplementation. In addition, the two supplements could reduce cardiac apoptotic markers by modulating cardiac Ob-R and antioxidant parameters. Moreover, the correlation of cardiac Ob-R and oxidative stress markers with the expression of the apoptotic proteins was noticed in the hearts of the T2DM male rats. Finally, we demonstrated *L*. *plantarum* accompanied by inulin as an effective useful supplement that may have beneficial effects on cardiac apoptosis inhibition in T2DM. Further studies are warranted to obtain more comprehensive results.

## Figures and Tables

**Table 1:** Composition of the high-fat diet (HFD).

Composition	Percentage
Powdered NPD	42
Cholesterol	1
Ghee	25
Sucrose	15
Flour	15
Cholic acid	0.5
Pea flour	0.5

**Table 2:** Diet and intervention from the beginning to the end of the study.

Markers	HFD	STZ injection	Normal diet	Saline gavage	Probiotic gavage	Prebiotic intake	Prebiotic and prebiotic intake
HC	—	—	Weeks 1 to 13	—	—	—	—
DC	Weeks 2 to 5	End of the week 5	Weeks 6 to 13	—	—	—	—
DSh	Weeks 2 to 5	End of the week 5	Weeks 6 to 13	Weeks 6 to 13	—	—	—
DL	Weeks 2 to 5	End of the week 5	Weeks 6 to 13	—	Weeks 6 to 13	—	—
DI	Weeks 2 to 5	End of the week 5	Weeks 6 to 13	—	—	Weeks 6 to 13	—
DLI	Weeks 2 to 5	End of the week 5	Weeks 6 to 13	—	—	—	Weeks 6 to 13

HC: healthy control; DC: diabetic control; DSh: diabetic sham; DL: diabetics treated by *L*. *plantarum*; DI: diabetics treated by inulin; DLI: diabetics treated by *L*. *plantarum* and inulin; T2DM: type 2 diabetes mellitus; BDNF: brain-derived neurotrophic factor; HFD: high-fat diet; STZ: streptozotocin.

**Table 3:** Correlation coefficients of food intake, FBS, and serum insulin with the expression of cardiac TNF-*α* and apoptotic proteins at the last week of the study.

	TNF-*α*/*β*-actin	FasL/*β*-actin	Cleaved caspase-9/procaspase-9	Cleaved caspase-8/procaspase-8	Cleaved caspase-3/procaspase-3
Food intake (week 13)	0.698^∗^	0.746^∗^	0.786^∗^	0.682^∗^	0.619^∗^
FBS	0.570^∗^	0.740^∗^	0.684^∗^	0.682^∗^	0.751^∗^
Serum insulin	-0.576^∗^	-0.755^∗^	-0.639^∗^	-0.759^∗^	-0.781^∗^

TNF-*α*: tumor necrosis factor-*α*; FasL: Fas ligand; BW: body weight; FI: food intake; FBS: fasting blood glucose. *P* based on the Pearson correlation test (*n* = 35). *P* < 0.05 was regarded as statistically significant. ^∗^*P* < 0.001.

**Table 4:** .Effects of *L*. *plantarum* and inulin supplementation on cardiac oxidative stress markers in T2DM rats.

Markers	HC	DC	DSh	DL	DI	DLI
Cardiac TAC (nmol/mg pro)	3.76 (0.40)	1.57 (0.18)^##^	1.35 (0.31)^##^	2.91 (0.40)^∗∗∗^	3.21 (0.56)^∗∗∗^	3.36 (0.43)^∗∗∗^
Cardiac SOD (U/mgr pro)	6.03 (0.88)	3.65 (1.15)^##^	3.61 (0.64)^##^	5.26 (0.93)^∗^	5.75 (0.25)^∗∗^	5.67 (0.38)^∗∗^
Cardiac GPx (U/gr pro)	0.37 (0.04)	0.16 (0.02)^##^	0.16 (0.03)^##^	0.30 (0.07)^∗∗^	0.25 (0.06)	0.30 (0.06)^∗∗^
Cardiac MDA (nmol/mg pro)	0.13 (0.01)	0.25 (0.02)^#^	0.23 (0.03)^#^	0.17 (0.06)	0.21 (0.07)	0.14 (0.03)^∗^

Data are expressed as means (SD). HC: healthy control; DC: diabetic control; DSh: diabetic sham; DL: diabetics treated by *L*. *plantarum*; DI: diabetics treated by inulin; DLI: diabetics treated by *L*. *plantarum* and inulin; TAC: total antioxidant capacity; SOD: superoxide dismutase; GPx: glutathione peroxidase; MDA: malondialdehyde.^##^*P* < 0.001, as compared to the HC group.^#^*P* < 0.01 as compared to the HC group. ^∗∗∗^*P* < 0.001 as compared to the DSh group. ^∗∗^*P* < 0.01 as compared to the DSh group. ^∗^*P* < 0.05 as compared to the DSh group.

**Table 5:** Correlation coefficients of cardiac oxidative stress markers and TNF-*α* with cardiac apoptotic markers.

Cardiac markers	FasL/*β*-actin	Cleaved caspase-9/procaspase-9	Cleaved caspase-8/procaspase-8	Cleaved caspase-3/procaspase-3
Cardiac TAC	-0.802^∗∗^	-0.873^∗∗^	-0.756^∗∗^	-0.708^∗∗^
Cardiac SOD	-0.673^∗∗^	-0.711^∗∗^	-0.618^∗∗^	-0.576^∗∗^
Cardiac GPx	-0.761^∗∗^	-.0727^∗∗^	-0.717^∗∗^	-0.737^∗∗^
Cardiac MDA	0.663^∗∗^	0.712^∗∗^	0.583^∗∗^	0.616^∗∗^
TNF-*α*/*β*-actin	0.614^∗∗^	0.767^∗∗^	0.662^∗∗^	0.529^∗^

TAC: total antioxidant capacity; SOD: superoxide dismutase; GPx: glutathione peroxidase; MDA: malondialdehyde; TNF-*α*: tumor necrosis factor-*α*; FasL: Fas ligand. *P* based on the Pearson correlation test (*n* = 35). *P* < 0.05 was regarded as statistically significant. ^∗∗^*P* < 0.001 and ^∗^*P* < 0.01.

## Data Availability

The datasets used and/or analyzed during the current study available from the corresponding author on reasonable request.

## Abbreviations

Bax: Bcl-2-associated X protein
CAT: Catalase
DCM: Diabetic cardiomyopathy
ER: Endoplasmic reticulum
FasL: Fas ligand
GPx: Glutathione peroxidase
GSH: Glutathione
HFD: High-fat diet
MDA: Malondialdehyde
Ob-R: Leptin or obesity receptor
ROS: Reactive oxygen species
SOD: Superoxide dismutase
STZ: Streptozotocin
T2DM: Type 2 diabetes mellitus
TAC: Total antioxidant capacity
TNF-*α*: Tumor necrosis factor-alpha.
